# Validity and Predictors of Medical Claims Coding for the Identification of Patients with Obesity

**DOI:** 10.36469/001c.164460

**Published:** 2026-08-20

**Authors:** Effie L. Kuti, Emma Richard, Kevin Schott, Christopher L. Crowe, Vincent Willey, Bonnie Donato

**Affiliations:** 1 Boehringer Ingelheim Pharmaceuticals Inc., Ridgefield, Connecticut, USA; 2 Carelon Research Inc., Wilmington, Delaware, USA; 3 Boehringer Ingelheim, Ridgefield, Connecticut, USA

**Keywords:** claims, coding, patients with obesity

## Abstract

**Background:**

The evolving obesity treatment landscape requires examining clinical and economic outcomes, but ICD-10-CM coding inconsistencies hinder patient identification in claims databases.

**Objectives:**

This study aims to validate obesity diagnosis codes and identify factors influencing their utilization.

**Methods:**

This retrospective cohort study utilized claims and clinical data from the Healthcare Integrated Research Database (HIRD®) to identify adults with a BMI of 30 kg/m² or higher, spanning the period from June 2021 to October 2022. The study examined the use of ICD-10-CM diagnosis codes for obesity across two distinct timeframes: a 6-month period before and after the index date for prevalence and modeling purposes, and a 60-day window around the index date for validity calculations. For validation, the sensitivity, specificity, positive predictive value (PPV), and negative predictive value (NPV) of the ICD-10-CM codes were measured, classifying patients as true or false positives or negatives based on their BMI and coding presence. Multivariable logistic regression was used to identify factors predicting obesity coding, guided by clinical reasoning, statistical results, and refined through backward elimination regression.

**Results:**

Out of 2 218 115 patients with available BMI data during the relevant period, 966 427 (44%) had a BMI of 30 kg/m² or above. The study identified 380,606 true positives, 17 422 false positives, 1 234 266 true negatives, and 585 821 false negatives, resulting in a PPV of 95.6%, a NPV of 67.8%, a specificity of 98.6%, and a sensitivity of 39.4%. The prevalence of obesity coding was 54%. Increased likelihood of coding was associated with being female, over the age of 24, non-White, insured by Medicare Advantage, having lower socioeconomic status, higher BMI, and receiving weight management interventions.

**Conclusion:**

ICD-10-CM codes demonstrate high specificity and PPV but low sensitivity, indicating that while coded cases are likely true obesity, many patients with obesity are not captured in claims.

## INTRODUCTION

Obesity has emerged as a pervasive public health issue since the late 1980s. As of 2018, 42% of adults in the United States (US) had a body mass index (BMI) greater than or equal to 30 kg/m^2^.^1^ Obesity is commonly categorized by BMI into class I obesity (30.0-34.9 kg/m^2^), class II obesity (35.0-39.9 kg/m^2^), and class III obesity (≥40.0 kg/m^2^), reflecting increasing levels of obesity severity.^2^ Obesity is associated with a spectrum of chronic conditions including, but not limited to, chronic kidney disease (CKD), hypertension, coronary artery disease, heart failure, stroke, type 2 diabetes (T2D), and metabolic dysfunction-associated steatohepatitis (MASH).^3,4^ Obesity is also associated with increased rate of progression of these conditions and increased risk of all-cause mortality; each 5-unit increase in BMI is associated with a 31% rise in risk of premature death.^5^ From an economic perspective, obesity poses significant financial challenges, with the US healthcare system spending $260.6 billion in 2016 on obesity-related costs, which equates to around $2505 per person with obesity.^6^ Addressing obesity, therefore, has implications for both public health and healthcare spending.

The treatment landscape for obesity care is rapidly evolving, giving patients and providers more treatment options. The American Society for Metabolic and Bariatric Surgery now advises bariatric surgery for all patients with a BMI of 35 kg/m^2^ or more, regardless of other health conditions.^7^ Furthermore, the Food and Drug Administration has approved the use of three glucagon-like peptide-1 receptor agonists (GLP-1 RA) for chronic weight management.^8^ These medications are now options for patients with a BMI ≥30 kg/m^2^ or 27 ≥kg/m^2^ with a weight-related comorbidity like CKD or T2D. These new care options, characterized by improved safety and effectiveness compared to prior weight management treatments, mark a significant development in the way providers and patients manage obesity.

In turn, the payer landscape for managing obesity is rapidly evolving. As commercial and public insurers’ as well as employer coverage policies for these new obesity treatments evolve, there is a pressing need to explore the clinical and economic outcomes associated with these treatments. However, there are uncertainties surrounding the completeness and accuracy of documentation of obesity through *International Classification of Diseases, Tenth Revision, Clinical Modification* (ICD-10-CM) coding in medical claims, a common real-world data source fit-for-purpose for cost and outcome evaluations. Specifically, there is lack of clarity around how often and for whom obesity ICD-10-CM diagnosis codes are used in medical claims. This ambiguity impacts accurate obesity patient identification in claims databases. Consequently, this affects the ability to effectively study this population and to identify the full population of patients eligible for chronic weight loss interventions, like bariatric surgery and GLP-1 RAs. Therefore, this study aims to validate obesity diagnosis codes in medical claims data and determine the factors that influence their use.

## METHODS

### Study Design and Data Source

This retrospective cohort study used data from the Healthcare Integrated Research Database (HIRD®), a large, geographically diverse healthcare database containing administrative claims for over 80 million people from commercial and Medicare Advantage/Supplemental health insurance plans in the Northeastern, Southern, Midwestern, and Western regions of the US since 1 January 2006. Clinical data from integrated electronic health records (EHR), including BMI, were available for 12% of patients in the HIRD over the period of interest. This study identified patients with a BMI ≥30 kg/m^2^ (ie, with obesity per the World Health Organization and the Centers for Disease Control) during the intake period, defined as 1 June 2021 to 30 October 2022.^2,9^ The intake period was selected to maximize use of the most contemporary data available at the time of analysis and to capture a period in which novel pharmacologic treatments for obesity management were in use in clinical practice.^8^ The index date was defined as the first observed BMI during the intake period, and therefore the date varied by patient. The first BMI measurement was used to anchor the analysis at the earliest available documentation of obesity status in the EHR, helping to avoid using future information and ensuring that baseline characteristics, coding assessments, and follow-up periods were properly aligned over time. Two baseline periods were employed in this study: A 6-month pre-index period, defined as the 6 months prior to the index date, and a full pre-index period, defined as any time prior to the index date going back to the earliest data available data using ICD-10-CM diagnoses codes (1 October 2015). The 6-month pre-index period was used to ensure the capture of medication use and healthcare encounters most proximal to identification of obesity. The full pre-index period was used to ensure the comprehensive capture of chronic conditions.

Two time periods were also employed to explore the presence or absence of ICD-10-CM coding for obesity among patients identified with BMI ≥30 kg/m^2^ This broader window was selected to reflect real-world variability in coding practices and healthcare utilization, recognizing that diagnosis codes may not be recorded at the same time as BMI measurement but rather during nearby clinical encounters. In contrast, a narrower 60-day window before and after the index date was used for validity calculations (sensitivity, specificity, positive predictive value [PPV], and negative predictive value [NPV]) to more closely align coding with the timing of BMI documentation in the EHR. This shorter window was intended to reduce temporal misalignment between measured obesity status and coding. However, because both windows include time after the index date, there is potential for temporal ambiguity, as some diagnosis codes may reflect documentation occurring after the BMI measurement rather than contemporaneously. These design choices represent a balance between capturing real-world coding practices and maintaining temporal proximity for validation.

### Study Population and Cohorts

The study population consisted of adult patients with at least 1 BMI measurement available in the HIRD during the intake period. Eligible patients were continuously enrolled in medical and pharmacy benefits for at least 6 months prior to the index date. Patients with an index BMI ≥30 kg/m^2^ were stratified into 2 subgroups, patients with and without an ICD-10-CM code for obesity in medical claims in the 6 months prior to or after the index date. Obesity was identified using ICD-10-CM diagnosis codes E66.0, E66.01, E66.09, E66.1, E66.2, E66.8, and E66.9, along with BMI Z-codes Z68.3-Z68.4. Female patients with diagnoses indicative of pregnancy within 9 months prior to or after the index date were excluded from analyses. Patients with BMI <30 kg/m^2^ were included solely to enable calculation of specificity and NPV but were excluded from regression analyses focused on predictors of coding among patients with obesity.

### Variables and Analyses

Demographics were evaluated as of the index date. Baseline comorbidities were assessed over the full pre-index period by the presence of ≥1 medical claim with a diagnosis for the comorbidity of interest. Baseline medication use and healthcare encounters were assessed over the 6-month pre-index period by the presence of ≥1 pharmacy claim for the medication of interest and ≥1 medical claim for health service of interest, respectively. Missingness was minimal for most variables given the use of administrative claims data. Race and ethnicity were derived using a proprietary multi-source algorithm with high completeness. For variables with missing values, missingness was retained as a separate category, and no imputation was performed.

To validate ICD-10-CM codes for obesity, sensitivity, specificity, PPV, and NPV were calculated. Each eligible patient was categorized into one of four categories: true positives (TP), false positives (FP), true negatives (TN), and false negatives (FN). A patient was considered a TP if their index BMI was ≥30 kg/m^2^ and they had ≥1 ICD-10-CM diagnosis codes for obesity around the index date (ie, 60 days prior to or after index date). A FP was defined as a patient with an index BMI <30 kg/m^2^, who had ≥1 ICD-10-CM diagnosis codes for obesity within the designated window. TNs, on the other hand, were patients whose BMI was <30 kg/m^2^ and had no ICD-10-CM diagnosis codes for obesity within the designated window. Finally, FNs were defined as those patients whose BMI was ≥30 kg/m^2^ but who had no ICD-10-CM diagnosis codes for obesity within the designated window. Sensitivity analyses were not performed and represent an area for future research.

The prevalence of ICD-10-CM coding for obesity was examined among all eligible patients with an index BMI ≥30 kg/m^2^ (ie, with obesity), as well as within subgroups characterized by different clinical characteristics (eg, with and without hypertension over the 6-month pre-index period). The denominator for these calculations was the total number of eligible patients with a BMI ≥30 kg/m^2^ and characteristic of interest. In contrast, the numerator comprised only those patients who also had a code for obesity within the designated window around the index date (ie, 6 months prior to or after index date).

Multivariable logistic regression was implemented to explore predictors of the presence or absence of ICD-10-CM codes for obesity within this designated window among patients with an index BMI ≥30 kg/m^2^. Candidate variables were selected a priori based on clinical relevance and prior literature, as well as descriptive analyses conducted within the study population. These variables included demographic characteristics (eg, age, sex, race/ethnicity, insurance type, and socioeconomic status), clinical characteristics (eg, BMI category, comorbid conditions), medication use, and healthcare utilization measures. To reduce model overfitting and improve interpretability, a backward elimination approach was applied, with variables retained in the final model based on statistical significance (*P*<.05) and clinical relevance. Multicollinearity among covariates was assessed using variance inflation factors, with a threshold of >10 indicating potential collinearity concerns. Model performance was evaluated using measures of discrimination and calibration. Discrimination was assessed using the C statistic (area under the receiver operating characteristic curve), while calibration was evaluated using the Hosmer–Lemeshow goodness-of-fit test. These diagnostics were used to ensure adequate model fit and stability of estimates. All covariates were measured during the pre-index period.

## RESULTS

### Study Population

This study identified 2 218 115 eligible patients with a BMI measurement in the HIRD during the intake period, with 966 427 (44%) having a BMI ≥30 kg/m^2^. Of these patients with obesity, 523 165 (54%) had a medical claim with an ICD-10 diagnosis code for obesity within 6 months before or after index date. Patients having a medical claim code with an ICD-10-CM code for obesity demonstrated distinct differences in demographic and clinical characteristics compared with those without a code (**Table 1; Supplementary Table S1**). On average, patients with a code were older at index date (53.6 vs 51.3 years) and were more likely to be insured through Medicare Advantage/Supplemental as opposed to commercial insurance (22% vs 16%) compared with those without a code. They also had a higher mean BMI (37.6 vs 34.6). Comorbidity burden, measured using the mean Quan Charlson Comorbidity Index, was also higher in this group (1.6 vs 1.2). Coded patients also had a greater proportion of individuals with each comorbidity of interest over the full pre-index period and each medication and healthcare service of interest over the 6-month pre-index period. Across all eligible patients, the most common comorbidities included hypertension, dyslipidemia, and lower back pain; the most frequently prescribed medications included antihypertensives, antihyperlipidemics, and antidepressants; and the most common healthcare encounters were visits to a cardiologist, physical therapy, and orthopedic encounters.

**Table 1.** Demographics and Clinical Characteristics Among Patients with a BMI ≥30 kg/m^2^ in EHR

	**Medical Claims Code (N = 523 165)**	**No Medical Claims Code (N = 443 262)**
Demographics		
Age in years, mean (SD)	53.6 (14.6)	49.7 (16.0)
Female, %	61	55
Insurance type, %		
Commercial	76	82
Medicare Advantage	22	16
Unknown/other	2	2
Race, %		
American Indian or Alaska Native, NH	1	<1
Asian, NH	1	2
Black, NH	15	12
Hispanic	6	6
Native Hawaiian or other Pacific Islander, NH	<1	<1
White, NH	75	77
Unknown/other	2	3
BMI, kg/m^2^, %		
Mean (SD)	37.6 (6.7)	34.6 (5.1)
Obesity class I (30-34.9)	44	66
Obesity class II (35-39.9)	28	22
Obesity class III (≥40)	28	12
Healthcare encounters, %		
Endocrinologist visit	6	3
Cardiologist visit	18	11
Bariatric surgery	2	<1
Orthopedic encounter	13	8
PT visit	13	10
Comorbidities, %		
QCI, mean (SD)	1.6 (2.1)	1.2 (1.9)
Anxiety disorder	41	36
Dyslipidemia	66	55
Hypertension	67	53
Lower back pain	49	42
OSA	29	17
Osteoarthritis of knee	23	16
T2D	33	22
Medication use, %		
Antihyperlipidemic medication	35	27
Antihypertensive medication	49	36
Chronic weight management medication	1	<1
GLP-1 RA for T2D	6	3

Abbreviations: BMI, body mass index; GLP-1 RA, glucagon-like peptide-1 receptor agonist; EHR, electronic health record; NH, non-Hispanic; OSA, obstructive sleep apnea; PT, physical therapy; QCI, Quan-Charlson Comorbidity Index; SD, standard deviation; T2D, type 2 diabetes.

### Validity of Codes

In the evaluation of the validity of obesity diagnosis codes, a total of 380 606 TP, 17 422 FP, 1 234 266 TN, and 585 821 FN were identified (**Table 2**). This distribution yielded a PPV of 95.6%, which implies that when a code for obesity is identified, there is a 95.6% probability that the patient’s BMI is ≥30 kg/m^2^. The NPV was recorded as 67.8%, indicating that if there is no observed code for obesity, the patient has a 67.8% chance of having a BMI <30 kg/m^2^. Specificity was 98.6%, suggesting that when a patient’s BMI is <30 kg/m^2^, there is a 98.6% likelihood that there will be no code for obesity. However, the sensitivity was found to be 39.4%, meaning that when a patient’s BMI is ≥30 kg/m^2^, there is only a 39.4% chance that an obesity diagnosis code will be present within 60 days before or after BMI documentation in the EHR. This relatively lower sensitivity points towards potential missed or underdocumentation of obesity in patients with a BMI ≥30 kg/m^2^.

**Table 2.** Validation of ICD-10-CM Obesity Coding Using BMI ≥30 kg/m^2^ as Reference Standard

	**ICD-10-CM Obesity Code Present**	**ICD-10-CM Obesity Code Absent**	**Total**
BMI ≥30 kg/m^2^ (obesity)	True positive: 380 606	False negative: 585 821	966 427
BMI <30 kg/m^2^	False positive: 17 422	True negative: 1 234 266	1 251 688
Total	398 028	1 820 087	2 218 115

Abbreviations: BMI, body mass index; ICD-10-CM, *International Classification of Disease, Tenth Revision–Clinical Modification*.

### Prevalence and Predictors of Coding

Overall, the prevalence for obesity coding in medical claims within 6 months before or after BMI documentation of obesity in the EHR was found to be 54%. The prevalence of obesity coding exhibited a wide range, from 39% to 88%, depending on demographic and clinical characteristics (**Table 3**). Coding prevalence was lowest among patients in the age groups 18-24 years (39%) and 25-34 years (46%), as well as those identified as obesity class I (44%). The highest prevalence of obesity coding was found among patients receiving chronic weight management medication (88%), those who underwent bariatric surgery (86%), and those receiving diet counseling and surveillance (84%). However, these groups represented less than 1%, 1%, and 3% respectively of patients with a BMI ≥30 kg/m^2^. Further examination revealed that among patients exhibiting the most common clinical characteristics described above, obesity coding prevalence were slightly higher but did not vary appreciably from the mean (58%-65%).

**Table 3.** Prevalence of ICD-10-CM Medical Claims Coding Among Patients with a BMI ≥30 kg/m^2^ in EHR

	**Prevalence, % (95% CI)**
Age, years	
18-24	38.7 (38.3-39.2)
25-34	46.3 (46.0-46.6)
35-44	52.5 (52.2-52.7)
45-54	55.8 (55.6-56.0)
55-64	56.3 (56.1-56.4)
65-74	60.1 (59.8-60.3)
≥75	52.9 (52.6-53.3)
BMI kg/m^2^	
Obesity class I (30-34.9)	44.0 (43.9-44.2)
Obesity class II (35-39.9)	59.8 (59.6-60.0)
Obesity class III (≥40)	73.9 (73.7-74.1)
Clinical characteristics	
Dyslipidemia	Present: 58.8 (58.6-58.9); absent: 46.9 (46.7-47.1)
Hypertension	Present: 59.9 (59.8-60.0); absent: 45.2 (45.1-45.4)
Lower back pain	Present: 57.8 (57.7-58.0); absent: 51.1 (50.9-51.2)
Antidepressant medication	Present: 59.9 (59.7-60.1); absent: 52.1 (52.0-52.2)
Antihyperlipidemic medication	Present: 60.5 (60.3-60.7); absent: 51.2 (51.1-51.4)
Antihypertensive medication	Present: 61.3 (61.2-61.5); absent: 48.7 (48.6-48.3)
Chronic weight management medication	Present: 87.6 (86.6-88.6); absent: 54.0 (53.9-54.1)
Bariatric surgery	Present: 85.8 (85.2-86.5); absent: 53.8 (53.7-53.9)
Diet counseling and surveillance	Present: 65.3 (65.0-65.5); absent: 52.2 (52.1-52.3)
Orthopedic encounter	Present: 63.2 (62.9-63.4); absent: 53.0 (52.9-53.1)
PT visit	Present: 60.8 (60.5-61.1); absent: 53.3 (53.1-53.4)

Abbreviations: BMI, body mass index; CI, confidence interval; EHR, electronic health record; PT, physical therapy.

**Figure 1.**
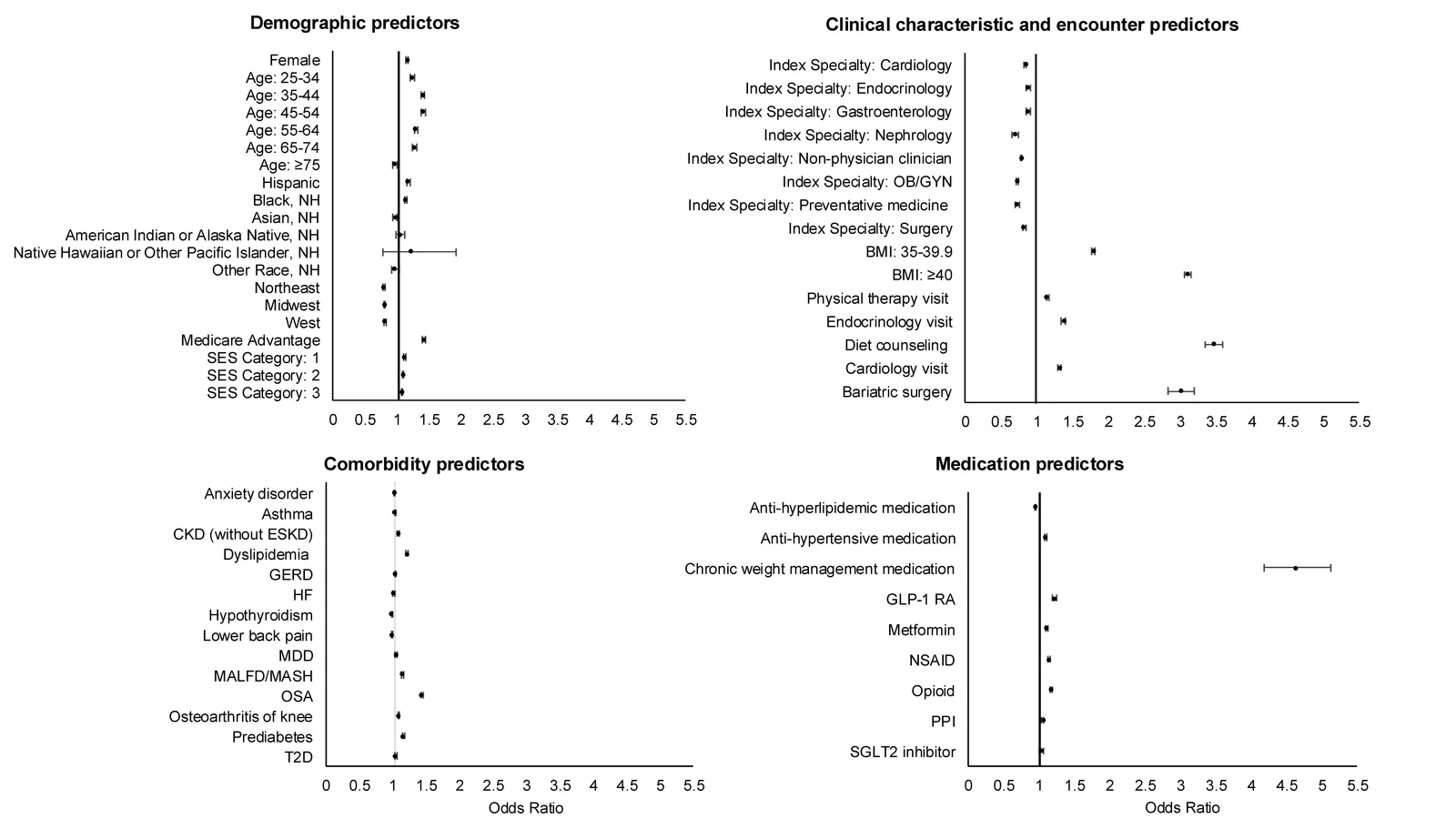
Logistic Regression Model Exploring Predictors of Medical Claims Coding for Obesity Among Patients with BMI ≥30 kg/m^2^ in the EHR Abbreviations: BMI, body mass index; CKD, chronic kidney disease; EHR, electronic health record; ESKD, end stage kidney disease; GERD, gastroesophageal reflux disease; GLP-1 RA, glucagon-like peptide-1 receptor agonist; HF, heart failure; MAFLD, metabolic dysfunction-associated fatty liver disease; MASH, metabolic dysfunction-associated steatohepatitis; MDD, major depressive disorder; NH, Non-Hispanic; NSAID, nonsteroidal anti-inflammatory drug; OSA, obstructive sleep apnea; PPI, proton pump inhibitor; SGLT2, sodium-glucose cotransporter-2; SES, socioeconomic status; T2D, type 2 diabetes). Reference groups: Sex, male; age, 18-24; race/ethnicity, White, NH; health plan product, commercial; region, South; SES category, 4; index provider specialty, primary care provider; BMI, 30-34.9; comorbidities, absent; medications, no use. Note: SES category 1 is lowest quartile and 4 is highest quartile.

The study found that certain clinical characteristics increased the likelihood of having a code for obesity (**Supplementary Table S2**). These included being female, being older than 24 years (up to age 74), identifying as non-White, being insured by Medicare Advantage, and having lower socioeconomic status, higher BMIs, and the presence of most comorbidities, medications, and encounters included in the model (**Figure 1**). Nevertheless, the impact of most of these clinical characteristics was modest with odds ratios (OR) close to 1, implying the increase in coding odds was not meaningful. However, receiving weight management interventions and having a BMI ≥35 kg/m^2^ considerably increased the odds of obesity coding, consistent with prevalence estimates. Specifically, patients who received chronic weight management interventions had over three times the odds of being coded for obesity, including bariatric surgery (OR, 3.01; 95% CI, 2.83-3.20), diet counseling and surveillance (OR, 3.47; 95% CI, 3.35-3.60), and receiving weight management medication (OR, 4.63; 95% CI, 4.19-5.13). Additionally, markedly higher BMI, specifically within obesity class III, compared with class I, tripled the odds of coding for obesity (OR, 3.11; 95% CI, 3.07-3.15).

## DISCUSSION

The present real-world study observed a contemporary cohort of almost 1 million patients with obesity based on BMI to assess coding of the condition in medical claims. The study evaluated the demographic and clinical characteristics of these patients and calculated the prevalence of obesity coding among patients with and without these characteristics. Furthermore, it ascertained the validity of the ICD-10-CM codes for obesity. Finally, it investigated predictors of coding among patients with a BMI ≥30 kg/m^2^. The research undertaken enhances our understanding of obesity identification in real-world healthcare settings using medical claims data.

Compared with those without obesity diagnosis codes in their medical claims within 6 months before or after documentation of a BMI indicative of obesity in the EHR, patients with diagnosis codes displayed higher proportions of comorbid conditions, medication usage, and healthcare interactions across all measurable variables. The observed trend of patients with codes having on average a higher comorbidity burden could be attributed to various factors. One potential explanation is that these patients may be under the care of healthcare providers and practices who are more conscientious in their medical billing coding practices. In such instances, these patients might appear relatively “sicker” when compared with those under the care of less accurate/comprehensive coders. An alternative explanation is that patients grappling with more severe health conditions generally experience more frequent touchpoints with the healthcare system.^10,11^ These increased encounters with healthcare providers enhance opportunities for patients’ conditions to be adequately identified and coded. Thus, the prevalence of coding might be reflective of the patients’ frequent interaction with the healthcare system. Importantly, these differences should be interpreted as predictors of obesity documentation and coding behavior, rather than predictors of obesity itself, since all patients in this comparison had BMI-defined obesity.

The results of this study underscore that if an obesity code is observed in a patient’s medical claims, we can confidently presume that the patient has a BMI of ≥30 kg/m^2^, as suggested by the high PPV of 95.6%. This finding corroborates earlier estimates by Ammann et al and Suissa et al, who reported high PPVs of 92.4% and 97.3%, respectively, reinforcing the accuracy of recording obesity codes in medical claims for patients diagnosed with obesity.^12,13^ However, the absence of an obesity code in the medical claims does not conclusively guarantee that a patient has a BMI <30 kg/m^2^. This indicates that obesity remains undercoded, with only a 39.4% (sensitivity) chance that a patient with a BMI ≥30 kg/m^2^ will have a code for obesity within 60 days prior to or after the observed BMI measure. Compared with previous studies investigating ICD-10-CM codes for obesity, our findings concur with the commonly observed trend of undercoding in the medical claims (sensitivity <40%).^12–14^ These observations suggest that reliance on codes alone is not sufficient to accurately identify the full obesity population, and that a substantial number of individuals thought not to have obesity would be miscategorized. This misclassification greatly limits the ability to perform research that assesses the prevalence and incremental burden attributed to obesity in a claims database. In addition, based on current medical claims coding practices, a significant segment of the population with obesity would likely be overlooked if coding was the sole method used to identify patients with obesity from a claims database. This highlights the importance of complementary methods of identifying cases of obesity.

These findings have important implications for studies that rely on administrative claims data to identify patients with obesity. Although the high PPV suggests that patients with an obesity diagnosis code are very likely to have BMI-defined obesity, the low sensitivity indicates that claims-based coding alone may substantially underidentify the full obesity population. As a result, studies using obesity diagnosis codes as the sole method of cohort identification may underestimate obesity prevalence and may select a subset of patients with greater clinical complexity, higher healthcare utilization, or more frequent obesity-related treatment. This has implications for health economics and outcomes research, including estimates of disease burden, healthcare resource utilization, treatment patterns, costs, treatment eligibility, and unmet need. Whenever possible, claims-based studies of obesity should consider supplementing diagnosis codes with additional data sources, such as EHR-derived BMI, procedure codes, medication claims, or validated algorithms, to improve case identification.

Undercoding of obesity appears consistent across patients regardless of most clinical characteristics. The prevalence of obesity coding did not show noticeable variation between patients with and without associated clinical characteristics, and no specific clinical characteristic notably increased the odds of coding. This suggests that coding discrepancies exist across all clinical subgroups, with no group more or less likely to have accurate obesity documentation. The issues with medical claims coding, thus, appear to be relatively evenly spread across all groups. However, higher coding prevalence was seen among patients receiving obesity-related services, including bariatric surgery, diet counseling and surveillance, chronic weight management medication, and among patients with higher BMI values. These findings may reflect, in part, greater clinical recognition or documentation of obesity when it is directly relevant to treatment planning, medical necessity, or coverage requirements.^15–17^ These findings are also consistent with broader literature suggesting that reimbursement policies and payment models may influence diagnostic coding accuracy across a variety of medical conditions.^18–21^ However, because the present study did not directly evaluate provider billing behavior, reimbursement policies, or payer-specific documentation requirements, the observed differences in obesity coding should be interpreted as associations rather than evidence of a causal effect of reimbursement incentives. Future studies could more directly evaluate whether coverage requirements, risk-adjustment programs, or clinical documentation workflows influence obesity coding practices.

Study findings should be interpreted in light of several limitations. Results may not generalize beyond commercially insured and Medicare Advantage populations or to individuals without similar insurance coverage. BMI is an imperfect proxy for adiposity and may misclassify obesity status because it does not directly measure body composition, body fat percentage, fat distribution, or individual health risk.^2,9^ This limitation may be particularly relevant across racial and ethnic groups, as prior research has shown that BMI-adiposity relationships can vary by race/ethnicity; therefore, a single BMI cutoff may not reflect the same level of adiposity or cardiometabolic risk across all populations.^22^ Because EHR-derived BMI was available only for a subset of HIRD patients, selection bias is possible if patients with BMI data differ systematically from those without it. Claims data may also contain coding omissions or errors and may incompletely capture sample medications, medications filled but not taken, health conditions not recorded in billing data, and unmeasured confounders. Overall, these limitations may affect generalizability and could lead to under- or overestimation of coding validity and predictors of obesity documentation.

## CONCLUSION

The undercoding of obesity in claims may contribute to underestimation of the population with obesity, affecting research, policymaking, resource allocation, and prevention and disease management strategies. In this study, ICD-10-CM codes demonstrated high specificity and PPV, suggesting that patients with an obesity code are likely to have BMI-defined obesity. However, the low sensitivity indicates that reliance on diagnosis codes alone is insufficient to identify the full population with obesity. More complete identification, particularly through approaches combining claims data with complementary information such as EHR-derived BMI, may improve population-level estimates and support more accurate assessments of treatment patterns, healthcare resource use, and obesity-related costs. Future research should continue to evaluate methods for identifying obesity in real-world data beyond ICD-10-CM codes alone.

### Disclosure

The authors did not receive payment related to the development of the manuscript. Boehringer Ingelheim was given the opportunity to review the manuscript for medical and scientific accuracy as well as intellectual property considerations. The study was supported and funded by Boehringer Ingelheim. Carelon Research, which is under contract from Boehringer Ingelheim. Carelon Research and Boehringer Ingelheim contributed to the conception of the work, study design, and interpretation of results, and drafting and review of the manuscript for medical and scientific accuracy. Carelon Research was responsible for statistical analysis and had access to patient-level study data; Boehringer Ingelheim did not have access to patient-level study data. All authors approved the final manuscript.

### Conflict of Interest

The authors declare no conflicts of interest regarding the publication of this paper.

## Supplementary Material

Online Supplementary Material

## Data Availability

Data are not available due to privacy and confidentiality considerations.
