# Supplementary material for: Validity and Predictors of Medical Claims Coding for the Identification of Patients with Obesity
Source: J Health Econ Outcomes Res. 2026 Aug 20;13(2):87–93. doi: 10.36469/001c.164460 (PMC13499625; doi:10.36469/001c.164460)
Supplement: Supplementary file 1 [file jheor_2026_13_2_164460_360620.pdf]

Journal of Health Economics and Outcomes Research

Online Supplementary Material

Validity and Predictors of Medical Claims Coding for the Identification of Patients with Obesity. *JHEOR*. 2026;13(2):87-93. doi:10.36469/jheor.2026.164460

Table S1: Complete Demographic and Clinical Characteristics Among Patients with BMI ≥30 kg/m² in the EHR

Table S2: Predictors of Medical Claims Coding for Obesity Among Patients with BMI ≥30 kg/m² in EHR

This supplementary material has been provided by the authors to give readers additional information about their work.

This is an open-access article distributed under the terms of the Creative Commons Attribution 4.0 International License (CCBY-4.0). View this license's legal deed at http://creativecommons.org/licenses/by/4.0 and legal code at http://creativecommons.org/licenses/by/4.0/legalcode for more information.

Table S1. Complete Demographic and Clinical Characteristics Among Patients with BMI ≥30 kg/m^2 in the EHR

	**Medical Claims Code (N = 523 165)**	**No Medical Claims Code (N = 443 262)**
Demographics		
Age in years, mean (SD)	54 (14.6)	51 (16.0)
Age category, %		
18-24 years	3	6
25-34 years	8	11
35-44 years	16	16
45-54 years	23	22
55-64 years	27	25
65-74 years	16	13
≥75 years	7	7
Female, %	61	55
Insurance type, %		
Commercial	76	82
Medicare Advantage	22	16
Unknown/other	2	2
Race, %		
American Indian or Alaska Native, NH	1	<1
Asian, NH	1	2
Black, NH	15	12
Hispanic	6	6
Native Hawaiian or Other Pacific Islander, NH	<1	<1
White, NH	75	77
Unknown/other	2	3
SES Index from lowest (1) to highest (4), %		
1	19	15
2	26	24
3	27	28
4	22	27
Missing	5	6
Index provider specialty, %		
Cardiology	5	4
Endocrinology	2	1
Gastroenterology	1	1
Nephrology	1	1
Nonphysician clinician	19	19
OB/GYN	5	6
PCP	29	25
Preventative medicine	1	1
Surgery	6	5
Unknown/other	31	39
BMI, kg/m^2		
Mean (SD)	38 (6.7)	35 (5.1)

Table S1. Complete Demographic and Clinical Characteristics Among Patients with BMI ≥30 kg/m² in the EHR

	**Medical Claims Code (N = 523 165)**	**No Medical Claims Code (N = 443 262)**
BMI category, %		
30-34.9	44	66
35-39.9	28	22
≥40	28	12
Healthcare encounters, %		
PT visit	13	10
Endocrinologist visit	6	3
Diet counseling and surveillance	4	1
Cardiologist visit	18	11
Orthopedic encounter	13	9
Bariatric surgery	2	<1
Comorbidities		
Anxiety disorder	41	36
Asthma	21	17
CKD, without ESKD	17	12
Dyslipidemia	66	55
GERD	41	32
HF	11	7
Hypertension	67	53
Hypothyroidism	23	19
Lower back pain	49	42
MDD	29	22
MAFLD/MASH	14	9
OSA	29	17
Osteoarthritis of knee	23	16
Prediabetes	19	13
T2D	33	22
Medication use, %		
Antidepressant medication	29	23
Antihyperlipidemic medication	35	27
Antihypertensive medication	49	36
Chronic weight management medication	1	<1
GLP-1 RA for T2D	6	3
Metformin	15	9
NSAID	21	15
PPI	19	13
SGLT2i	4	2

Abbreviations: BMI, body mass index; CKD, chronic kidney disease; EHR, electronic health record; ESKD, end stage kidney disease; GERD, gastroesophageal reflux disease; GLP-1 RA, glucagon-like peptide-1 receptor agonist; HF, heart failure; MAFLD, metabolic dysfunction-associated fatty liver disease; MASH, metabolic dysfunction-associated steatohepatitis; MDD, major depressive disorder; NH, Non-Hispanic; NSAID, nonsteroidal anti-inflammatory drug; OB/GYN, obstetrics and gynecology; OSA, obstructive sleep apnea; PCP, primary care provider; PPI, proton pump inhibitor; PT, physical therapy; SGLT2, sodium-glucose cotransporter-2; SES, socioeconomic status; T2D, type 2 diabetes).

Table S2. Predictors of Medical Claims Coding for Obesity Among Patients with BMI ≥30 kg/m^2 in EHR

	OR	95% LCI	95% UCI	*P* Value
Demographics and social determinants of health				
Female (reference: male)	1.16	1.14	1.17	≤.001
Age in years (reference: 18-24)				
25-34	1.24	1.21	1.28	≤.001
35-44	1.40	1.37	1.43	≤.001
45-54	1.41	1.38	1.45	≤.001
55-64	1.30	1.27	1.33	≤.001
65-74	1.27	1.24	1.31	≤.001
≥75	0.97	0.93	1.00	.041
Race/ethnicity (reference: white, NH)				
Hispanic	1.17	1.15	1.20	≤.001
Black, NH	1.13	1.12	1.15	≤.001
Asian, NH	0.97	0.94	1.00	.082
American Indian or Alaska Native, NH	1.05	0.99	1.12	.094
Native Hawaiian or other Pacific Islander, NH	1.23	0.78	1.92	.374
Other, NH	0.97	0.92	1.03	.325
Unknown	0.97	0.94	1.01	.136
Region (reference: South)				
Northeast	0.79	0.78	0.81	≤.001
Midwest	0.80	0.79	0.81	≤.001
West	0.81	0.80	0.83	≤.001
Unknown/other	0.88	0.81	0.95	≤.001
Health plan product (reference: commercial)				
Medicare Advantage	1.42	1.39	1.44	≤.001
Unknown/other	0.81	0.78	0.84	≤.001
Index specialty (reference: PCP)				
Cardiology	0.84	0.82	0.86	≤.001
Endocrinology	0.89	0.85	0.92	≤.001
Gastroenterology	0.88	0.85	0.92	≤.001
Nephrology	0.70	0.66	0.75	≤.001
Nonphysician clinician	0.79	0.78	0.80	≤.001
OB/GYN	0.73	0.72	0.75	≤.001
Preventative medicine	0.73	0.70	0.76	≤.001
Surgery	0.83	0.81	0.85	≤.001
Unknown/other	0.61	0.61	0.62	≤.001
SES category (reference: 4, highest)				
1 (lowest)	1.12	1.10	1.13	≤.001
2	1.09	1.08	1.11	≤.001
3	1.08	1.07	1.09	≤.001
Unknown/other	1.08	1.06	1.10	≤.001
BMI category (reference: obesity class I [30-34.9])				
Obesity class II, 35-39.9)	1.79	1.77	1.81	≤.001
Obesity class III, ≥40)	3.11	3.07	3.15	≤.001
Comorbidities during full pre-index period				
Anxiety disorder	1.03	1.01	1.04	≤.001
Asthma	1.04	1.03	1.05	≤.001

Table S2. Predictors of Medical Claims Coding for Obesity Among Patients with BMI ≥30 kg/m² in EHR

	OR	95% LCI	95% UCI	*P* Value
CKD (without ESKD)	1.09	1.07	1.10	≤.001
Dyslipidemia	1.22	1.20	1.23	≤.001
GERD	1.04	1.03	1.05	≤.001
HF	1.02	1.00	1.04	≤.001
Hypothyroidism	0.99	0.98	1.00	0.030
Lower back pain	0.99	0.98	1.00	0.044
MDD	1.05	1.04	1.06	0.012
MAFLD/MASH	1.15	1.13	1.16	≤.001
OSA	1.43	1.42	1.45	≤.001
Osteoarthritis of the knee	1.09	1.08	1.10	≤.001
Prediabetes	1.16	1.15	1.18	≤.001
T2D	1.04	1.03	1.06	≤.001
Medications during 6-month pre-index period				
Anti-hyperlipidemic medication	0.96	0.95	0.97	≤.001
Anti-hypertensive medication	1.10	1.09	1.12	≤.001
Chronic weight management medication	4.63	4.19	5.13	≤.001
GLP-1 RA	1.22	1.19	1.26	≤.001
Metformin	1.12	1.10	1.14	≤.001
NSAID	1.14	1.13	1.16	≤.001
Opioid	1.18	1.16	1.19	≤.001
PPI	1.06	1.04	1.07	≤.001
SGLT2 inhibitor	1.03	1.01	1.07	0.014
Encounters during 6-month pre-index period				
Bariatric surgery	3.01	2.83	3.20	≤.001
Cardiology visit	1.32	1.30	1.34	≤.001
Diet counseling and surveillance	3.47	3.35	3.60	≤.001
Endocrinology visit	1.38	1.34	1.41	≤.001
PT visit	1.15	1.14	1.17	≤.001

Abbreviations: BMI, body mass index; CKD, chronic kidney disease; EHR, electronic health record; ESKD, end stage kidney disease; GERD, gastroesophageal reflux disease; GLP-1 RA, glucagon-like peptide-1 receptor agonist; HF, heart failure; MAFLD, metabolic dysfunction-associated fatty liver disease; MASH, metabolic dysfunction-associated steatohepatitis; MDD, major depressive disorder; NH, Non-Hispanic; NSAID, nonsteroidal anti-inflammatory drug; OB/GYN, obstetrics and gynecology; OR, odds ratio; OSA, obstructive sleep apnea; PCP, primary care provider; PPI, proton pump inhibitor; PT, physical therapy; SGLT2, sodium-glucose cotransporter-2; SES, socioeconomic status; T2D, type 2 diabetes).
